# Thirty-Nine Years Later: A Case Report and Literature Review of Delayed Pleural and Pericardial Effusions After Chest Radiotherapy for Non-Hodgkin Lymphoma

**DOI:** 10.7759/cureus.80528

**Published:** 2025-03-13

**Authors:** Laxman Wagle, Anuj Timshina, Hom N Pant, Vikas Pathak

**Affiliations:** 1 Internal Medicine, Ascension Saint Agnes Hospital, Baltimore, USA; 2 Internal Medicine, MedStar Franklin Square Medical Center, Baltimore, USA; 3 Internal Medicine, Patan Academy of Health Science, Kathmandu, NPL; 4 Interventional Pulmonology and Critical Care Medicine, Virginia Institute of Lung Diseases, Mechanicsville, USA

**Keywords:** delayed radiotherapy-related pleural effusions, non-hodgkin lymphoma, radiation-induced lung injury, radiation-induced pleural effusions, radiotherapy related pericardial effusion

## Abstract

Radiation therapy (RT) is a common treatment for non-Hodgkin lymphoma (NHL) but can lead to long-term pulmonary and cardiovascular complications. Delayed radiotherapy-related pleural effusion (DRPE) and pericardial effusion are rare sequelae, with few cases reported. This case highlights recurrent pleural and pericardial effusions nearly 40 years after chest RT, underscoring the need for ongoing surveillance in cancer survivors.

A 51-year-old female with a history of nodular sclerosing NHL in remission after RT in 1977 presented in 2016 with recurrent bilateral pleural and pericardial effusions. Despite multiple interventions, including pericardiocentesis, thoracenteses, and pleural catheter placement, her effusions persisted. An extensive workup ruled out malignancy, infection, and autoimmune causes, ultimately attributing the effusions to radiation-induced lung injury. Despite ongoing management, she was discharged to hospice care.

DRPE is a diagnostic challenge due to its delayed onset, sometimes appearing decades after RT. It can present with variable pleural fluid characteristics. Radiation-induced lung injury is a known complication of thoracic RT, with risk factors including radiation dose and pre-existing pulmonary conditions. Management is symptomatic, with treatments such as NSAIDs, corticosteroids, diuretics, and pleural drainage, though outcomes vary. In this case, pleural catheter placement offered temporary relief, but recurrent effusions led to hospice care.

This case highlights the need for long-term monitoring in survivors of chest RT, as delayed pulmonary and cardiovascular toxicities can arise decades later. Given the rarity of DRPE and the lack of standardized treatment, further research into protective strategies and early interventions for radiation-induced lung injury is essential to improve cancer survivors' quality of life.

## Introduction

The standard approach for treating non-Hodgkin lymphoma (NHL) typically combines systemic chemotherapy with radiation therapy (RT). Recent advancements focus on using lower doses and more focused irradiation, known as involved-field irradiation, to reduce the risk of severe side effects [1]. However, RT is widely used in managing various cancers, including lymphoma, but it may result in thoracic, cardiovascular, and pulmonary complications such as pneumonitis, pleuritis, and pericarditis [2]. The phenomenon of patients developing pneumonitis after active treatments have been completed is called radiation recall [3]. Radiation-related lung damage encompasses conditions such as bronchial stenosis, pulmonary edema, pleural effusions, fibrosis, and pneumonitis. Aqeel et al. conducted a retrospective analysis of 96 cancer patients receiving thoracic irradiation, revealing that 53% developed pleural effusions, 19% of which were classified as radiation-induced pleural effusions (RIPE), predominantly ipsilateral to the irradiated area (67%), with a median onset of six months (four to eight months) [4]. Delayed pleural effusion following radiation, known as delayed radiotherapy-related pleural effusion (DRPE), is a rare condition with only a few cases reported in the literature.

This case report describes the rare occurrence of recurrent pericardial and bilateral pleural effusions in a 51-year-old female with nodular sclerosing NHL, in remission, 39 years after completion of RT to the neck and thorax.

## Case presentation

A 51-year-old female with a history of nodular sclerosing NHL in remission, following extensive RT to the neck and thorax in 1977, presented with recurrent pleural and pericardial effusions. Her medical history was significant for hypothyroidism secondary to thyroidectomy, osteoporosis, atypical lymphoid infiltrative skin lesions, and a distant history of deep vein thrombosis in the 1980s while on oral contraceptives. In 2016, she presented to the emergency department with worsening shortness of breath and was found to have a large pericardial effusion with tamponade physiology, along with bilateral pleural effusions. She denies smoking or illicit drug use. She appeared alert and thin on physical examination, with a BMI of 17.91 kg/m². There was no lymphadenopathy or heart murmurs, and a lung examination revealed absent breath sounds at the base with no other remarkable findings. Initial pertinent laboratory findings are in Table 1 below.

**Table 1:** Initial pertinent laboratory findings including leukocytosis with microcytic anemia, hyponatremia, hyperkalemia, and hypochloremia WBC: white blood cell, Hgb: hemoglobin, MCV: mean corpuscular volume, PLT: platelet, Na: sodium, K: potassium, Cl: chloride, M: male, F: female

Test	Result	Reference range	Interpretation
WBC	14.6 × 10³/µL	4.0-11.0 × 10³/µL	Leukocytosis
Hgb	8.9 g/dL	13.5-17.5 g/dL (M)/12.0-15.5 g/dL (F)	Anemia
MCV	69 fL	80-100 fL	Microcytosis
PLT	417 × 10³/µL	150-400 × 10³/µL	Thrombocytosis
Na	127 mmol/L	135-145 mmol/L	Hyponatremia
K	5.7 mmol/L	3.5-5.1 mmol/L	Hyperkalemia
Cl	94 mmol/L	98-107 mmol/L	Hypochloremia

**Figure 1:**
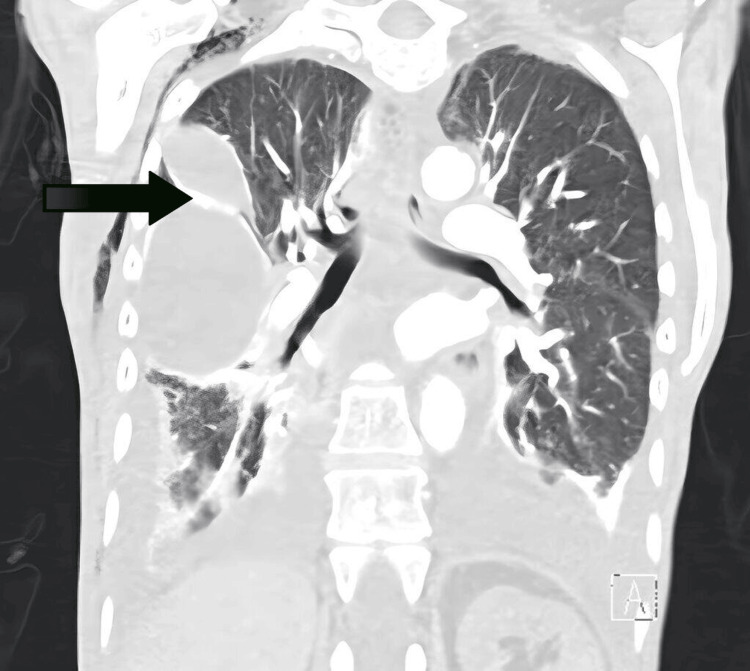
CT showing complex large loculated pleural effusion in the right side (arrow) CT: computed tomography

Over the next 18 months, she underwent multiple interventions to manage these recurrences. These included pericardiocentesis for tamponade and bilateral thoracenteses for persistent pleural effusions. Due to the chronic nature of her effusions, left- and right-sided indwelling pleural catheters were eventually placed for drainage. The patient had extensive evaluations to determine the etiology of her pleural and pericardial effusions, which remained unclear. During her treatment course, serial imaging, including chest CT scans, demonstrated moderate to large loculated pleural effusions (Figure 1) and a small to moderate pericardial effusion. However, there was no evidence of empyema or pulmonary embolism. Pleural fluid analyses were predominantly transudative with lymphocyte predominance, while multiple cytological and microbiological studies were negative for malignancy or infection. A pleural biopsy showed fibrotic tissue with acute and chronic inflammatory changes but no evidence of malignancy or specific infection. Rheumatologic testing, including Sjögren's anti-SS-A and SS-B, antinuclear antibodies (ANA), anti-dsDNA, rheumatoid factor (RF), and extended autoimmune panel, was negative, ruling out autoimmune causes. PET did not show hypermetabolic adenopathy. A medical thoracoscopy was attempted but was aborted due to bleeding, and subsequent video-assisted thoracoscopic surgery revealed no additional findings.

The patient’s effusions were ultimately attributed to radiation-induced damage from her childhood cancer treatment 39 years earlier. Due to refractory effusions despite ongoing interventions, she required multiple hospitalizations. Ultimately, she was discharged to hospice care.

## Discussion

Radiation-induced lung injury (RILI) affects 5-20% of patients, leading to dyspnea and chronic lung fibrosis [5]. RILI has two phases: radiation pneumonitis, an inflammatory phase occurring one to three months after RT, and radiation fibrosis, a scarring phase developing six to 24 months after RT [6]. RILI presents with symptoms like dyspnea, dry cough, and fever. The physical exam is often normal, but signs including pleural friction rub and rales may be rarely present [6]. Due to nonspecific symptoms and signs, diagnosis is clinical, making it challenging.

Zhao et al. found that RIPE occurred in 29.1% of patients (51 cases) [7]. RIPE developed at a median of 3.7 months (0.6-18.0 months) after thoracic RT [7]. Among 40 symptomatic patients, 60% experienced chest pain, 30% had a cough, and 45% reported dyspnea or shortness of breath. The actuarial incidence of RIPE was 88.8% at one year and 11.2% at two years [7]. Most patients receiving thoracic RT are at risk of RILI, but factors like smoking history, chronic obstructive pulmonary disease, and interstitial lung disease can further increase the risk [5]. Increased RIPE risk correlated with dosimetric factors like mean lung dose [8-10]. A typical lung dose of 7 Gy is linked to symptomatic pneumonitis, with an occurrence rate of 5% [11].

An interesting finding from Aqeel et al.'s study is that 44% of RIPE patients also had concurrent radiation pneumonitis or pericardial effusion, highlighting the simultaneous involvement of the cardiovascular and respiratory systems, similar to our case [4]. Radiation-induced heart disease can develop years after RT, leading to valve, pericardial, coronary, muscle, or conduction problems [12]. Cancer survivors following RT should have annual checkups, screenings for radiation-induced heart disease, and symptom assessments [2]. A healthy lifestyle with a balanced diet, exercise, weight control, and no smoking is essential [13]. High-risk survivors may need a baseline echocardiogram 6-12 months after RT [13].

DRPE often presents with atypical clinical symptoms and signs, with a highly variable interval between radiation exposure and its onset. Therefore, diagnosing DRPE requires a pleural biopsy and diagnostic tests to rule out other malignancies, infections, and autoimmune causes. Comprehensive rheumatologic testing is essential, including Sjögren's antibodies (anti-SS-A and SS-B), ANA, anti-dsDNA, and RF. Bernardeschi et al. described a rare recurrent benign pleural effusion as the sole manifestation of Hodgkin disease, localized to the lateral thoracic wall. The diagnosis was confirmed through CT imaging and needle biopsy, with no mediastinal or hilar lymph node involvement [14]. This case highlights the possibility of Hodgkin lymphoma recurrence presenting only as pleural effusion, which should be considered in similar scenarios. Table 2 shows the comparative analysis of reported DRPE cases.

**Table 2:** Comparative analysis of reported DRPE cases DRPE: delayed radiotherapy-related pleural effusion, NHL: non-Hodgkin lymphoma, LDH: lactate dehydrogenase, RT: radiation therapy

Study	Post-radiation period	Diagnosis	Effusion	Management
Rodríguez-García et al., 1991 [15]	8 years	Following mediastinal radiotherapy for Hodgkin disease.	Serohemorrhagic exudate without malignant cells, percutaneous biopsy showed multiple reactive mesothelial cells without neoplastic cells	Received 150 mg of indomethacin daily, following which symptoms improved dramatically, and the patient was discharged three weeks after entry
Fragoulis et al., 2006 [16]	23 years	Following radiation treatment for the nodular sclerosis type of Hodgkin disease	Likely exudative specific gravity of 1.020, protein of 4.6 g/dL, glucose of 98 mg/dL, pericardium biopsy showed a chronic inflammatory reactive process with no other neoplastic changes	Pericarditis and pleural effusion were not controlled with regimens including steroid and non-steroid anti-inflammatory agents. The fluid collections improved only with doxycycline (100 mg twice a day)
Katano et al., 2022 [17]	25 years	Thoracic RT for the treatment of stage IIA Hodgkin lymphoma	Exudative (protein of 4.5 g/dL, albumin of 3 g/dl, glucose of 112 mg/dl, LDH of 108 IU/L)	Periodic thoracentesis to relieve respiratory distress, followed by the oral administration of tolvaptan
Chotirmall et al., 2008 [18]	36 years	Following mediastinal radiotherapy for NHL	Transudative effusion (pH of 7.43, protein of <30 g/dL, albumin of <15 g/dl, glucose of 6.8 mmol/l, LDH of 211 IU/L)	Therapeutic drainage of 1 L was performed with no re-collection of fluid
Shen et al., 2018 [19]	25 years	Following radiotherapy for Hodgkin lymphoma	Transudative pleural effusion (protein of 3.07 g/dL, lactate dehydrogenase of 63.0 U/L)	Prednisolone at a daily dose of 30 mg was administered and titrated down as symptoms improved until the patient was discharged from our hospital
Current case	39 years	Following radiotherapy for NHL	Transudative pleural effusion, pleural biopsy showed fibrotic tissue with acute and chronic inflammatory change	PleurX catheters, pericardiocentesis. Given the recurrent nature of effusion, the patient was discharged to hospice care

The abovementioned table highlights the variability in DRPE presentations. Pleural fluid analysis in DRPE cases lacks a consistent pattern, with some showing an exudative profile while others are transudative. In our review, including this case, three were exudative, and three were transudative [15-19]. Pleural biopsy consistently demonstrates fibrotic tissue with acute and chronic inflammatory change. Treatment generally follows standard pleural effusion management, including diuretics, thoracentesis, indwelling pleural catheters, and pleurodesis. Since DRPE is rare, there is no established protocol for its diagnosis and management. This lack of awareness may also contribute to its underdiagnosis. Therapies, such as NSAIDs and prednisolone, have shown good results in managing DRPE because of their anti-inflammatory properties. Our patient also initially received prednisone, suspecting lupus, before the diagnosis of DRPE was confirmed. However, prednisolone was stopped after significant weight gain. Fragoulis et al. reported improvement with oral doxycycline in a case of delayed effusive pericarditis and recurrent pleural effusion after the failure of steroids and NSAIDs [16]. However, the required pleural fluid concentration and optimal dosage of doxycycline remain unclear. Corticosteroids have been effective in treating radiation-induced pericardial effusion [20]. Kumagai et al. reported a case of radiation-induced pleuritis in a patient with esophageal cancer, successfully managed with 30 mg of prednisolone, which controlled pleural fluid re-accumulation and was gradually tapered [21]. Shigematsu et al. described treating RIPE with diuretics and 16 mg of dexamethasone over six days, though it proved insufficient [22].

In cases like ours, where recurrent refractory pleural effusion persists despite multiple interventions, symptomatic management with pleural catheter placement and pleurodesis will be beneficial.

## Conclusions

Our case, the first to report recurrent pericardial and bilateral pleural effusions nearly 40 years after RT, underscores the importance of long-term monitoring in cancer survivors. While DRPE presents significant diagnostic challenges due to its variable fluid characteristics, careful differentiation from other causes, including malignancy, is essential. Although there is no standardized treatment for DRPE, management strategies such as NSAIDs, corticosteroids, diuretics, and pleural drainage can offer symptomatic relief. In our case, the recurrent nature of the effusions ultimately led to hospice care. However, the frequency and severity of such complications have changed with the advent of advanced imaging modalities, improved radiation techniques, and lower radiation doses in the modern era. These technological advancements necessitate a reevaluation of the clinical significance of late toxicities like DRPE in today’s cancer treatment landscape. This case highlights the need for ongoing surveillance, personalized management, and further research to mitigate long-term pulmonary and cardiovascular toxicities, ultimately enhancing the quality of life for survivors of RT.
